# Seasonal abundance and habitat use of bird species in and around Wondo Genet Forest, south‐central Ethiopia

**DOI:** 10.1002/ece3.2926

**Published:** 2017-04-04

**Authors:** Zerihun Girma, Yosef Mamo, Girma Mengesha, Ashok Verma, Tsyon Asfaw

**Affiliations:** ^1^ School of Wildlife and Eco‐tourismHawassa UniversityHawassaEthiopia; ^2^ Department of BiologyHawassa UniversityHawassaEthiopia

**Keywords:** altitude, average vegetation height, distribution, feeding guilds, migratory, slope

## Abstract

The habitat use and seasonal migratory pattern of birds in Ethiopia is less explored as compared to diversity studies. To this end, this study aimed at investigating the patterns of distribution related to seasonality and the effect of habitat characteristics (elevation, slope, and average vegetation height) on habitat use of birds of Wondo Genet Forest Patch. A stratified random sampling design was used to assess the avian fauna across the four dominant habitat types found in the study area: natural forest, wooded grassland, grassland, and agroforestry land. A point transect count was employed to investigate avian species richness and abundance per habitat type per season. Ancillary data, such as elevation above sea level, latitude and longitude, average vegetation height, and percent slope inclination, were recorded with a GPS and clinometers per plot. A total of 33 migratory bird species were recorded from the area, of which 20 species were northern (Palearctic) migrants while 13 were inter‐African migrants. There was a significant difference in the mean abundance of migratory bird species between dry and wet seasons (*t* = 2.13, *p* = .038, *df* = 44). The variation in mean abundance per plot between the dry and wet seasons in the grassland habitat was significant (*t* = 2.35, *p* = .051, *df* = 7). In most habitat types during both dry and wet seasons, omnivore birds were the most abundant. While slope was a good predictor for bird species abundance in the dry season, altitude and average vegetation height accounted more in the wet season. The patch of forest and its surrounding is an important bird area for migratory, endemic, and global threatened species. Hence, it is conservation priority area, and the study suggests that conservation coupled with ecotourism development is needed for its sustainability.

## 1 Introduction

Abundance of bird species is largely influenced by the spatiotemporal distribution of some key environmental resources (McCain, 2009). As a result, various studies elsewhere in the world attempted to study factors that affect bird abundance and distribution at spatial and temporal scales (Lincolin, Fredrick, Peterson, & Zimmerman, 1998; Mengesha, Mamo, & Bekele, 2011; Newton, 2008). Seasonality plays a major role in determining the abundance and distribution of birds. Seasonality affects food and cover availability of bird population, which in turn affects breeding success and ultimately survival of the bird species (Mengesha & Bekele, 2008). The seasonal variation in the amount of rainfall and temperature and spatial and temporal microhabitat conditions are known to affect the availability of various food items for birds (Mengesha et al., 2011). Based on species sensitivity to the type of habitat, these could alter the diversity, abundance, and distribution of birds in an area. Particularly, it has been revealed that processes acting in breeding and wintering grounds determine both the patterns of habitat occupancy and seasonal abundance in migratory bird species (Newton, 2008). Tropical and subtropical countries witness a certain type of seasonal migration of birds, which is not well known in the northern latitudes.

On the other hand, the spatial distributions of food and cover resources determine the abundance of bird species. The food and cover requirement of bird species is determined mainly by the vegetation structure and composition that is correlated with abundance and habitat use (Waterhouse, Mather, & Seip, 2002). In turn, environmental factors such as elevation, slope, and aspect determine the vegetation composition and structure, ultimately affecting bird species abundance and habitat use. Elevation and slope affect vegetation structure site productivity, distribution, composition, and secondary biotic interactions (Hofer, Bersier, & Felix, 1999; Waterhouse et al., 2002). Vegetation structure partly determines prey availability and seasonal migration of birds, ultimately determining bird community structure (Lincolin et al., 1998).

Various studies have attempted to model species–habitat relationships (McCain, 2009; Waterhouse et al., 2002). While many studies have documented patterns in diversity along elevation gradients and have attempted to describe the mechanisms underlying those patterns, the consensus on the generality of pattern and processes is still a topic of discussion (Rahbek, 2005). Most studies focused on how species richness and distribution vary among elevation gradients. Because elevation affects the condition of the physical environment and the kinds and amounts of resources available for breeding and foraging activities, the composition and structure of bird communities may change along elevation gradient (McCain, 2009; Mengesha et al., 2011; Rahbek, 2005). Particularly, it has been pointed out that as elevation increases, the availability of resources for birds diminishes reflecting differences in forest stand structure, site productivity, vegetation species composition, stand disturbance patterns, secondary biotic interactions, and available land area (Rahbek, 2005). Understanding such patterns and their underlying mechanisms is critically important for conservation efforts.

Ethiopia harbors 863 species of birds, of which 639 are resident and 224 are regular seasonal migrants, including 176 from the Palearctic and 48 inter‐African (Lepage, 2014, 2016). Lepage (2016) also indicated that 19 species are endemic to Ethiopia whereas 31 are globally threatened, 1 introduced species and a further 13 are shared only with Eritrea. Despite the rich bird assemblages in Ethiopia, due to enormous habitat degradation, fragmentation and loss their survivals of many bird species including the endemic and globally threatened ones are endangered (Lepage, 2014, 2016). Particularly, expansion of agriculture, livestock encroachment, deforestation, indiscriminate fire fuel by the ever increasing human population has been often cited as the major cause of birds habitat degradation, fragmentation and loss in Ethiopia ultimately affecting the survival of birds (Aynalem & Bekele, 2008; Mengesha et al., 2011). It has been indicated that threats to bird species of Ethiopia have been diagrammatically increasing in the past few decades, as it is true to other wildlife (Aynalem & Bekele, 2008).

Updated information on birds' abundance and habitat use in the present study area, Wondo Genet Forest, and its surrounding human‐modified habitat, although known to harbor about 111 bird species (Sim, 1979), is lacking. Most bird studies in Ethiopia focused on characterizing the birds species composition and abundance on specific regions of the country (Aynalem & Bekele, 2008; Mengesha & Bekele, 2008). Only few studies attempted to determine the seasonal abundance of bird species in relation to environmental variables such as vegetation species composition and structure (Mengesha et al., 2011). Studies on the seasonal abundance of birds along elevation, slope, and aspect gradients are rare, if any. Particularly, in the present study area, there had not been attempts to study the seasonal distribution and habitat use of bird species. However, the knowledge of seasonal abundance and habitat use of birds is central for monitoring of bird species targeted for conservation planning. Moreover, the knowledge of seasonal distribution and habitat use of birds is extremely important in areas where anthropogenic activities are major factors threatening the survival of the bird species. It has also been indicated that threats such as deforestation and livestock encroachments affect the abundance and distribution of birds as it affects the cover and food requirement (Mengesha et al., 2011). To this end, the present study aimed at investigating the seasonal abundance and distribution and determining the environmental factors that affect the habitat use of birds in and around Wondo Genet Forest.

## 2 Methods

### 2.1 Study area

**Figure 1:**
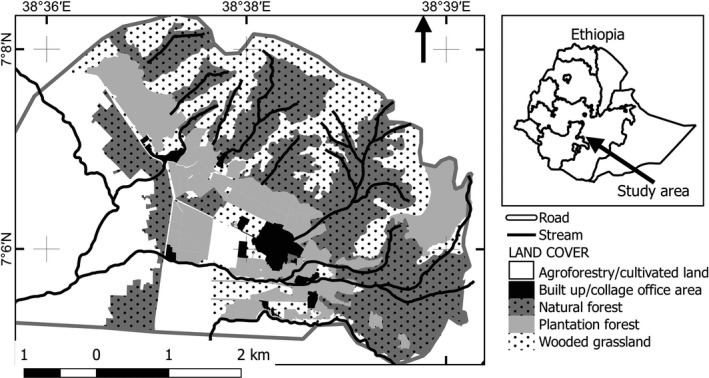
Location map of the study area (source: NASA Landsat Program, 2003, Landsat ETM+ scene L71168055_05520051203.ETMGLS2005, SLC‐Off, USGS, Sioux Falls, 03/12/2005)

The study area is situated in the southern part of Ethiopia at about 260 km south of the capital city, Addis Ababa, and located between 7°5′30″ and 7°7′30″ N latitude and 38°36′30″ and 38°39′0″ E longitude (Figure 1). The study was carried out in a remnant forest patches and mosaics of wooded grasslands, agroforestry land, grasslands, and built‐up areas covering a total of 958 hectares that is owned by Wondo Genet College of Forestry and Natural Resources (WGCFNR), Hawassa University. The lower elevation areas were mostly covered by agroforestry, the college infrastructure, and plantation forests and grasslands, while the uplands were covered by the natural forests and wooded grassland.

For this study, the area was stratified into four habitat types based on altitudinal ranges and vegetation types (Figure 1). Natural forest represents valley areas and middle altitude areas between 1,800 and 2,200 m asl with dominant tree species of *Celtis africana, Albizia guminefera, Croton macrostachus,* and *Millettia ferruginea* (Kebede, Yirdaw, Luukkanen, & Lemenih, 2013). The natural forest was relatively intact and undisturbed compared to other habitat types and was covered with tall giant dominant tree species mentioned above and few fruiting tree species such as *Ficus vasta* and *Ficus sur* in the lower altitudes (Kebede et al., 2013). The wooded grassland habitat covers the upland areas with rugged topography (2,200–2,670 m asl) and dominated by grassland intermixed with scattered stands of shrubs predominantly *Protea aguedi*,* Myyrsine africana, Dodonaea viscose*,* D*. *angustifolia*, and scattered *Erica arborea* at the summit of mount Abaro (Kebede et al., 2013). Anthropogenic fire occasionally occurs in the upper altitude of the wooded grassland habitat often impacting the vegetation dynamics and survival of the wildlife species (Girma, Mamo, & Ersado, 2012; Kebede et al., 2013). The grassland habitat consisted of undifferentiated grassland with scattered tress species of *Podocarpus falcatus*,* Acacia* spp., and *Eucalyptus* spp. and occurs from 1,760 to 1,780 m asl. The grassland habitat was disturbed habitat with livestock grazing and human encroachments. This habitat was managed by the college through prescribed burning and grass cutting in an attempt to grew fresh grass for the college's dairy livestock grazing, burning occurred during the study period. The agroforestry land consisted of coffee plantation and cultivated land covered by such as sugarcane plantation and vegetables which occupies the lower altitudes areas (1,747–1,775 m asl) of the study area.

The study area falls under humid montane climate having a bimodal rainfall during the long rainy season from June to October and the shorter rainy season occurring from March to April. The total amount of annual rainfall varies between 700 and 1,400 mm with an average of 1, 200 mm (Kebede et al., 2013). The mean monthly temperature is 19.5°C (Kebede et al., 2013).

### 2.2 Data collection and analysis

A stratified random sampling design across the four dominant habitat types found in the study area, natural forest, wooded grassland, grassland, and agroforestry land, was used following the methods of Jones (1998), Krebs (1999), and Aynalem and Bekele (2008). Location points were randomly generated in a geographical information system (GIS) using ArcGIS software v10.1 (ESRI, 2012). From field observations and site descriptions found in the literature (Girma et al., 2012; Kebede et al., 2013), the approximate area of each habitat type in the study area was estimated to determine the proportion of sample plots needed to represent each of the four habitat types. A total of 16 point transects representing each habitat were systematically established to estimate the diversity and abundance of birds of Wondo Genet Forest Patches. Six point transects were established in the natural forest, six in the wooded grassland, two in the grassland, and two in the agroforestry habitat. Navigation to each plot location was made using a Garmin eTrex Legend Global Positioning System (GPS). The radius of each point transect was 30 m. Each point transect was 100 m far away from road side to avoid edge effect and was at least 300 m far away from each other to avoid double counting of the same individual of a species following Aynalem and Bekele (2008).

Data collection was carried out from December 2011 to July 2012. According to the bimodal rainfall distribution of the area (Kebede et al., 2013), four months—December, January, February, and May—were considered as dry season, while March, April, June, and July were considered as wet season. Bird identifications and counting of individuals were conducted by direct observations aided with binoculars (7 × 400). Observations were made by standing in the middle of the point transect and observing 360° round quietly and gently up to a distance of 30 m radius. Observation at each point transect lasted for 15 min, and field guides of Perlo (1995) and Redman, Stevenson, and Fanshawe (2012) were used for identification of the birds. In each observation, bird species were identified and numbers of individuals observed within the 30 m radius were recorded on data sheet prepared for this purpose. Survey of the birds was carried out in the morning from 07:00 to 10:00 a.m. Each point transect was visited eight times in the course of the study period. In addition, ancillary data, such as elevation above sea level, latitude and longitude, average vegetation height, and percent slope inclination, were recorded with a GPS and clinometers per plot.

All data were summarized per plot per habitat types during both the dry and the wet season in a table. Relative abundance (%) = *n*/*N* × 100, where *n* is the number of individuals of particular species recorded and *N* is the total number of individuals of the species. Two‐sample *t*‐test was used to compare the mean species richness and abundance per habitat types per plot between dry and wet seasons. The stepwise regression analysis (backward elimination technique) was carried out on the species richness and abundance of birds for the 16 plots sampled as the outcome variable to evaluate parameters of the habitats (slope, elevation, and average vegetation height) that account for the birds' disproportionate habitat use. This was carried out for species richness and abundance determined during both the dry and wet seasons, and model selection was based on *F* and *p* values. Durbin–Watson statistic and VIF were used to examine multicollinearity of the predictors. Backward elimination continued until the “*minimum F‐to‐remove”* dropped below the specified probability level (0.1). All computations were performed using MINITAB 17 (Minitab Inc., 2013).

## 3 Results

### 3.1 Seasonal relative abundance

A total of 1,672 individuals grouped in to 137 bird species were recorded. Of the recoded bird species, one endemic and six near‐endemic (endemic to Ethiopia and Eretria) species were identified from the area (Table 1). Abyssinian dark‐headed oriole (*Oriolus monacha*), the near‐endemic, was relatively the most abundant bird species among the migratory and endemic bird species in the study area number 144 (24.5%). The endemic species yellow‐fronted parrot (*Poicephalus flavifrons*) was the third relatively abundant species among the migratory and endemic bird species in the area 62 (10.67%; Table 1). There were six globally threatened bird species recorded, of which three are rare/accidental, two endangered, and one near‐threatened according by IUCN red list (Table 1). Generally, there were more individuals of globally threatened bird species during dry season than during wet season (Table 1).

**Table 1:** Seasonal relative abundance of migratory, endemic, and globally threatened bird species in Wondo Genet Forest

Common name	Scientific name	Seasonal abundance				
		MS	Wet	Dry	Total	RA
Abyssinian dark‐headed oriole	*Oriolus monacha* a	RS	60	84	144	24.75
Barn swallow	*Hirundo rustica*	NM	26	44	70	12.05
Yellow‐fronted parrot	*Poicephalus flavifrons* b	RS	19	43	62	10.67
Wattled ibis	*Bostrychia carunculata* a	RS	6	51	57	9.81
Rock martin	*Hirundo fuligula*	NM	2	42	44	7.57
White‐backed vulture	*Gyps africanus* c	RS	2	28	30	5.16
Alpine swift	*Tachymarptis melba*	NM	7	20	27	4.65
African paradise flycatcher	*Terpsiphone viridis*	(AM)	5	14	19	3.27
Ruppell's vulture	*Gyps rueppellii* c	RS	8	6	14	2.4
Black‐winged lovebird	*Agapornis taranta* a	RS	7	6	13	2.23
Willow warbler	*Phylloscopus trochilus*	NM	2	8	10	1.72
Cyprus wheatear	*Oenanthe cypriaca* d	NM	0	8	8	1.38
Red‐chested cuckoo	*Cuculus solitarius*	(AM)	1	6	7	1.2
Tambourine dove	*Turtur tympanistria*	(AM)	3	4	7	1.2
Whinchat	*Saxicola rubetra*	NM	0	6	6	1.03
Yellow wagtail (flava)	*Motacilla flava*	NM	0	6	6	1.03
Abyssinian roller	*Coracias abyssinicus*	(AM)	0	4	4	0.69
Common quail	*Coturnix coturnix*	(AM)	0	4	4	0.69
Nyanza swift	*Apus niansae*	NM	0	4	4	0.69
Abyssinian salty flycatcher	*Melaenornis chocolatina* a	RS	1	2	3	0.52
Black cap	*Sylvia atricapilla*	NM	1	2	3	0.52
Cattle egret	*Bubulcus ibis*	(AM)	0	3	3	0.52
Northern carmine bee‐eater	*Merops nubicus*	NM	0	3	3	0.52
Red‐shouldered cuckoo shrike	*Campephaga flava*	(AM)	2	1	3	0.52
Ring‐necked dove	*Strepetopelia capicola*	(AM)	0	3	3	0.52
Levaillant's cuckoo	*Clamator levaillantii*	(AM)	2	0	2	0.34
Lilac‐breasted roller	*Coracias caudatus*	(AM)	0	2	2	0.34
Pied wheatear	*Oenanthe pleschanka*	NM	0	2	2	0.34
Pygmy sunbird	*Anthreptes platurus* d	RS	1	1	2	0.34
Red‐billed quelea	*Quelea quelea*	(AM)	1	1	2	0.34
Red‐rumped swallow	*Cecropis daurica*	(NM)	2	0	2	0.34
Tawny eagle	*Aquila rapax*	(NM)	0	2	2	0.34
White‐winged Cliff‐chat	*Thamnolaea semirufa* a	RS	2	0	2	0.34
Black‐billed wood dove	*Turtur abyssinicus*	(AM)	0	1	1	0.17
Black red start	*Phoenicurus ochruros*	NM	0	1	1	0.17
Common red start	*Phoenicurus phoenicurus*	NM	1	0	1	0.17
Ethiopian cisticola	*Cisticola lugubris* a	RS	1	0	1	0.17
Isabelline wheatear	*Oenanthe isabellina*	NM	1	0	1	0.17
Klaas's cuckoo	*Chrysococcyx klaas*	(AM)	0	1	1	0.17
Northern wheatear	*Oenanthe oenanthe*	NM	0	1	1	0.17
Pallid harrier hawk	*Circus macrourus* e	NM	0	1	1	0.17
Peregrine falcon	*Falco peregrinus*	(NM)	0	1	1	0.17
Siberian stone chat	*Saxicola maurus* d	RS	0	1	1	0.17
Wire‐tail swallow	*Hirundo smithii*	(NM)	0	1	1	0.17

MS, migratory status; AM, inter‐African migrant; (AM), inter‐African migrant with breeding subpopulation; NM, northern migrant; (NM), northern migrant with breeding subpopulation; RS, resident.

a Near‐endemic.

b Endemic.

c Endangered.

d Rare/accidental.

e Near‐threatened.

Of the total recorded birds, 33 were migratory species, of which 20 were northern (Palearctic) migrants and 13 were inter‐African (Table 1). During the dry season, most (29) migratory bird species were recorded, of which 17 species were northern migrants and 12 species were inter‐African migrants (Table 1). Among the migratory bird species recorded during the wet season (14), eight species were northern migrants and five were inter‐African migrants. In terms of number of individuals 196 individuals of migratory bird species were recorded during the dry season, while 56 individuals were recorded during the wet season. Of the 196 individuals recorded in the dry season, 152 (77%) individuals were northern migrants, while 44 (27%) individuals were inter‐African migrants. Of the 56 individuals of bird species recorded in the wet season, 42 (75%) were northern migrants, while 14 (25%) were inter‐African migrants. There was a significant difference in the mean relative abundance of migratory bird species between dry and wet seasons (*t* = 2.13, *p* = .038, *df* = 44).

### 3.2 Habitat use

The three habitat types (wooded grassland, grassland, and agroforestry) had more or less similar species richness of birds (Table 2), while lowest variation in species richness per plot between wet and dry seasons was recorded in the natural forest (Table 2). The highest variation in mean abundance per plot between dry and wet seasons was recorded in the grassland, while the least was recorded in the natural forest (Table 2). The variation in mean abundance per plot between the dry and wet seasons in the grassland habitat was significant (*t* = 2.35, *p* = .051, *df* = 7).

**Table 2:** Mean species richness and abundance per plot per season among different habitat types during both wet and dry seasons

	Agroforestry land		Grassland		Natural forest		Wooded grassland	
	Wet	Dry	Wet	Dry	Wet	Dry	Wet	Dry
MSRPP	4.5 ± 1.02	5.6 ± 0.56	5.5 ± 0.63	6.4 ± 1.41	3.5 ± 0.32	3.8 ± 0.41	4.2 ± 0.45	5.3 ± 0.52
MAPP	18.9 ± 6.41	20.1 ± 9.48	16.1 ± 1.83	35.6 ± 8.74	7.1 ± 0.71	7.2 ± 1.13	10.8 ± 1.98	14.8 ± 2.40

MSRPP, mean species richness per plot; MAPP, mean abundance per plot.

**Figure 2:**
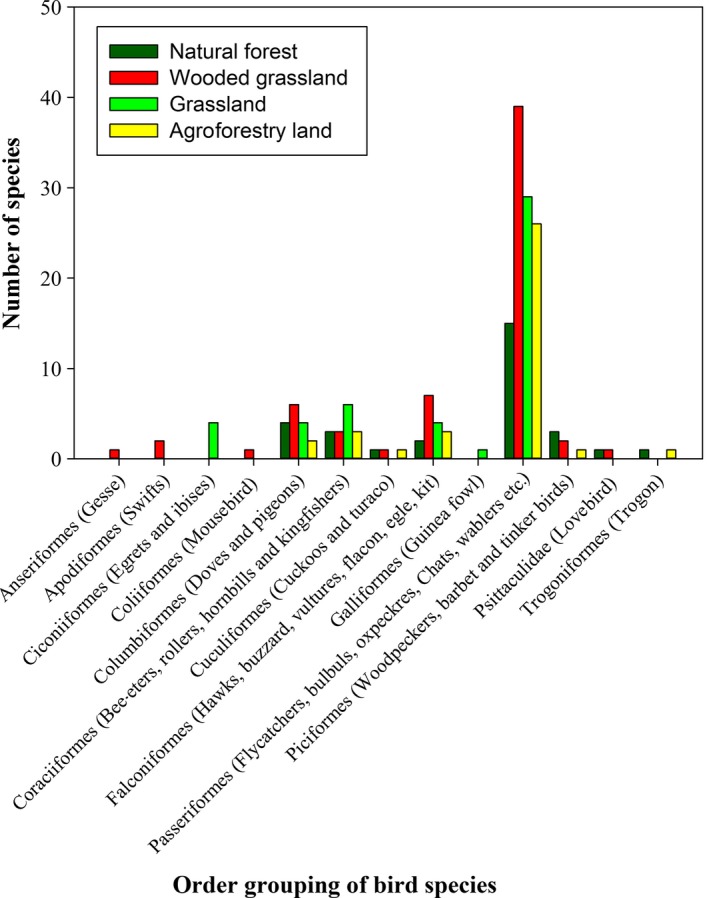
Feeding guilds of birds per season among four habitat types in Wondo Genet Forest

In most of the habitats, omnivore birds were abundant guild during both the dry and wet seasons, whereas the natural forest consisted of relatively highest number of omnivore species during both the dry and wet seasons (Figure 2). In grassland habitat, carnivores were relatively most abundant, especially during the dry season. Granivores were relatively abundant in the agroforestry habitat as compared to other habitat types during the dry season. Insectivores were relatively abundant in wooded grassland, grassland, and agroforestry habitat types especially during the dry season.

Six models were eventually fitted that quantitatively and qualitatively explain which of the habitat components accounted for habitat use by the bird species. Habitat quality varied for both species richness and abundance during the wet and dry seasons. While slope was a good predictor for bird species abundance in the dry season, altitude and average vegetation height accounted more in the wet season (Table 3). Average vegetation height accounted for total species richness variation in the study area, while both altitude and average vegetation height accounted for difference in total bird abundance in the study area (Table 3).

**Table 3:** Summary statistics for selected models that describe habitat use of birds in the dry and wet seasons. The Durbin–Watson statistic (D–W) and Variance Inflation Factor (VIF) were used to examine autocorrelation and multicollinearity of the predictor variables

Model	Habitat variable	Coefficient	*p*	VIF	*F*	Overall model			
						*p*	S	R (%)	D–W
BSRDS	Constant	5.054				.000	1.3	17.2	2.43
	Av. Veg. height	−0.108	.111	1.00	2.90				
BSADS	Constant	18.65				.000	6.87	30.5	2.3
	Slope	−0.56	.010	1.00	2.94				
	Av. Veg. height	−0.108	.111	1.00	2.93				
BSRWS	Constant	4.9				.000	1.12	30.5	2.37
	Av. Veg. height	−0.13	.027	1.00	6.14				
BSAWS	Constant	60.20				.001	6.36	63.0	2.12
	Av. Veg. height	−1.43	.001	1.1	20.1	.000			
	Altitude	−0.02	.019	1.1	7.23				
TSR	Constant	15.37				.001	1.45	52.1	1.84
	Av. Veg. height	−0.27	.003	1.1	13.95				
	Altitude	−0.003	.143	1.1	2.43				
TBA	Constant	106.8				.000	6.23	81.79	1.68
	Av. Veg. height	−2.18	.000	1.1	49.11				
	Altitude	−0.03	.000	1.1	25.06				

BSRDS, bird species richness dry season; BSADS, bird species abundance dry season; BSRWS, bird species richness wet season; BSAWS, bird species abundance wet season; TSR, total species richness; TBA, total bird abundance.

## 4 Discussion

The significant variation in abundance of migratory bird species between the dry and wet seasons could be due to seasonal movement patterns, local and regional habitat changes, large‐scale population changes, and climatic conditions (Aynalem & Bekele, 2008; Ericia, Den, Tom, & Meire, 2005; Gaston et al., 2000). The relatively higher numbers of near‐endemic species (around 50% of the country near‐endemic species) and globally threatened species (around 20% of country globally threatened species) in the area indicate that the mosaic landscape at varying altitude that consists of small patch of forest is important area of endemism and conservation priority area. Furthermore, it is known in Ethiopia that highland areas have more endemic species while lowlands consisted of high species diversity of both plants and animals, supporting the result of the study. The relatively higher abundance of the endemic yellow‐fronted parrot (*Poicephalus flavifrons*) and the near‐endemic Abyssinian dark‐headed oriole (*Oriolus monacha*) supports the fact that the area is important conservation priority area and potential for ecotourism (bird‐watching) that sustainably links economic gain with conservation. The occurrence of 33 migratory species also reveals the importance of the area to serve as sanctuary for wintering northern migrant birds and inter‐African migrants.

Generally, the results of this study indicate that the area despite its small size is a home to large number of bird species. This could be due to the uniqueness of the area in providing heterogeneous habitats for the birds. The area is known for its impressive only remnant forest in eastern escarpment of the rift valley (Kebede et al., 2013). The establishment of WGCFNR in the 1970s saved the forest and grasslands form destruction, while most of the forest and grasslands in the surrounding areas are destructed and converted to agricultural landscapes. Studies elsewhere in the world and in Ethiopia have revealed that deforestation due to expansion of agriculture altered the habitats of birds negatively impacting their abundance and distribution (Aynalem & Bekele, 2008; Mengesha et al., 2011; Pennington & Blair, 2011; Ukmar, Battisti, Luislli, & Bolongna, 2007). Furthermore, due to its close proximity to five rift valley lakes (which are known as sanctuary for migratory birds, especially Abijatta‐Shalla Lakes National Park), the remnant forest could serve as alternative habitat for the migratory species due to its uniqueness in vegetation composition and structure, which provide better foraging and nesting opportunities. The migratory species could use the area for resting, foraging (to store enough fat for the journey back home), and other activities while waiting for the favorable condition back their home range of residence (Manu, 2000). The relatively higher diversity of migratory bird species also signifies the area among the most important bird area (EWNHS, 1996).

The significant variation in bird species abundance could be due to the migratory behavior, the availability of food, habitat condition, and breeding season of the species (Mengesha & Bekele, 2008; Tilahun, Travi, & Valles, 2001). The results of this study indicated that the area is home to considerably high number of migratory species. For migratory birds, different processes acting in breeding and wintering grounds determine both the patterns of habitat occupancy and the effects of the consequent distribution of individuals on population dynamics (Holmes, Marra, & Sherry, 1996; Sherry & Holmes, 1996), affecting the seasonal abundance and distribution. Migratory birds are known to move between different geographical areas including making the longest migration from the Northern Hemisphere to Southern Hemisphere in response to change in climatic conditions that affect food and cover availability (Newton, 2008). The presence of Palearctic migrants in the present area migrating from temperate zone to tropical zone buttresses this idea. During winter period, due to severe snow formation the food and cover availability will be severely impacted and temperature fall below zero, in response to this, the birds are forced to migrate in warm tropical zones (Holmes et al., 1996; Sherry & Holmes, 1996). Furthermore, the birds avoid to bird in winter; in fact, some have breeding population in where they migrate (Aynalem & Bekele, 2008). Due to this reason, the abundance of birds in the study area during dry season (winter in temperate zone) could increase as there are new arrivals of bird species during dry season, most of which fly back to their resident home during wet season. The distinct seasonality of rainfall and seasonal variation in the abundance of food resources result in seasonal changes in the species abundance of birds (Gaston et al., 2000). The distribution and abundance of many bird species are determined by the composition of the vegetation that forms a major element of their habitats. It is known that the vegetation composition and structure is affected by rainfall patterns which change between wet and dry seasons. As vegetation changes along complex geographical and environmental gradients, a particular bird species may appear, increase or decrease in number, and disappear as the habitat changes (Lee & Rotenberry, 2005). However, the decrease in abundance of birds during the wet season could also be affected by the surrounding agricultural landscape habitat. During the wet season, the surrounding agricultural lands are covered by crops that provide alternative temporary seasonal foraging and nesting opportunities to the birds. This could decrease their abundance in their natural habitat during wet season (Aynalem & Bekele, 2008). However, during dry season as all the crops are harvested, they will leave no foraging and nesting opportunity for birds and hence could only be confined in the forested habitat. Furthermore, the wet and relatively cold weather could affect the behavior of the birds, restricting their movement and affecting the citing of the birds by the observers. The season availability of migratory species could signify the importance of the area as wintering habitats for migratory birds. However, the threats (expansion of agriculture, illegal settlement, deforestation, and livestock encroachments; Girma et al., 2012; Kebede et al., 2013) to the remnant patch of forest could further shrink the habitat availability of the birds impacting the survival of the migratory species, including some that are globally threatened.

The significant seasonal variation in mean abundance of birds in the grassland habitat could be due to the preference of grassland habitat over other habitat types by migratory bird species. As most of the migratory species were carnivore, insectivore, and granivore, they could have preferred the grassland habitat to easily pick their prey and grains for granivore species. It is known that insects, invertebrates, small mammals, and grains are more abundant in grassland habitat than in forested habitats (Shochat, Lerman, & Fernández‐Juricic, 2010). The relatively higher abundance of granivores in the agroforestry habit could be due to the abundance of cultivated crops such as maize and coffee. The higher abundance of omnivores in the natural forest could be as a result of the good availability of both animal and plant source diet in which omnivore bird species are evolutionarily adapted to feed (Mengesha et al., 2011). The results indicate that heterogonous habitats should be maintained, as the niche requirements of the birds vary based on species.

Primary topographic factors (e.g., slope, aspect, elevation) alter microclimate conditions and indirectly affect the growth and distribution of land cover, hence affecting bird distribution and abundance. Slope may act as an important input for microclimatic conditions affecting the growth and distribution of vegetation. Slope affects the amount of solar radiation received by vegetation, soil moisture, and microclimatic variables (Bennie, Huntleya, Wiltshirea, Hill, & Baxtera, 2008; Koenig, 2012), ultimately affecting birds' abundance and distribution. The relatively higher bird species richness and abundance in the lower elevations than higher elevations could be due to the heterogeneous nature of habitats in the lower altitude near human settlement areas that gives better foraging opportunities and diverse nesting and roosting sites. Various studies also documented a general decrease in species richness and abundance along an elevation gradient (e.g., McCain, 2009; Rahbek, 2005). Contemporary climate (temperature and rainfall), biological processes (mass effects, productivity, habitat heterogeneity, interspecific interactions, and evolutionary process), and historical processes (niche conservatism, isolation, speciation, endemism, and evolutionary diversification) are the common correlates of and drivers of elevation patterns of bird diversity (Hawkins, Diniz‐Filho, Jaramillo, & Soeller, 2007; Machac, Janda, Dunn, & Sanders, 2011).

The decrease in species abundance and richness as vegetation height increases could be as a result of a decrease in heterogeneity in habitat type, absence of sufficient fruiting trees, and risk of predation that could be higher in the natural forest. Other studies elsewhere have shown that habitat heterogeneity increases birds' abundance, and birds were more abundant in heterogeneous habitats near human settlement areas than the homogenous forest (Pennington & Blair, 2011; Shochat et al., 2010). Furthermore, it has been pointed out that bird abundance decreases with canopy cover closure in a well‐developed forest (McWethy et al.*,* 2009). Daniels (1989) also found that bird diversity was negatively correlated with canopy density but positively correlated with the coefficient of variation of canopy density suggesting that a uniform canopy has lesser number of bird species.

## 5 Conclusion

The remnant patch forest and its surrounding areas of Wondo Genet College campus is an important sanctuary for migratory species and home to endemic and near‐endemic species. Other habitat types such as grassland and agroforestry surrounding the patch of forest are also important habitats for high diversity of bird species including the migratory ones. The study also revealed that seasonality and habitat types are important in determining both the migrant and nonmigrant bird species abundance and distribution in the area indicating specific habitat use by some of the species. The different land use type had species in common and species specific to specific habitat in response to ecological factors such as altitudes, vegetation, and climate across the landscape of the area. It has been revealed in the results that birds' abundance is affected by the availability of food and cover, which is influenced mainly by vegetation composition and structure. However, the anthropogenic activities going on in and around the remnant forest can shrink the available habitats to birds through their action in altering the vegetation composition and structure that ultimately affects birds' abundance and survival. Hence, urgent conservation measures are inevitable to conserve the endemics and globally threatened bird species in the area. There is a need for biodiversity conservation integrating the remnant forest and other heterogonous habitats together with the surrounding agricultural landscape.

## Conflict of Interest

None declared.
